# Effectiveness of interactive dashboards to optimize prescribing in general practice: a systematic review

**DOI:** 10.1093/fampra/cmaf036

**Published:** 2025-06-04

**Authors:** Caroline McCarthy, Patrick Moynagh, Áine Mannion, Ashely Wei, Barbara Clyne, Frank Moriarty

**Affiliations:** Department of General Practice, RCSI University of Medicine and Health Sciences, Dublin 2, Ireland; Department of General Practice, RCSI University of Medicine and Health Sciences, Dublin 2, Ireland; Department of General Practice, RCSI University of Medicine and Health Sciences, Dublin 2, Ireland; Department of General Practice, RCSI University of Medicine and Health Sciences, Dublin 2, Ireland; Department of Public Health & Epidemiology, School of Population Health, RCSI University of Medicine and Health Sciences, Dublin 2, Ireland; School of Pharmacy and Biomolecular Sciences, RCSI University of Medicine and Health Sciences, Dublin 2, Ireland

**Keywords:** interactive dashboards, potentially inappropriate prescribing, audit and feedback, preventable drug-related morbidity, polypharmacy, comparative benchmarking, electronic health records, routine prescription data

## Abstract

**Background:**

The World Health Organisation’s Medication Without Harm campaign aims to reduce severe avoidable medication-related harm by 50%. This systematic review explored the characteristics of interventions that provide visual and longitudinal feedback on prescribing (interactive dashboards), in general practice and the effect of these interventions on prescribing-related outcomes.

**Methods:**

This systematic review was registered prospectively and reported in line with PRISMA guidelines. Multiple databases and grey literature were searched in November 2023 to identify interventional studies that explored the effect of interactive dashboards on prescribing-related outcomes in general practice. Two independent researchers conducted screening, data extraction, and risk of bias assessment. Interventions were described narratively, and a random-effects meta-analysis was performed for comparable studies.

**Results:**

Ten randomized controlled trials, one controlled before-and-after study, and three interrupted time series were included. Seven studies reported a significant positive effect on prescribing-related outcomes, with an effect seen more often for studies focussing on potentially inappropriate prescribing (PIP) (four out of six). Three of the eight studies that focussed on antibiotic prescribing demonstrated a significant effect. A meta-analysis of three RCTs involving 160 general practices and 198 135 patients demonstrated the overall odds of PIP was 0.92 (95%CI: 0.78–1.06, *I*^2^ = 70.1%) in the intervention compared to the control group.

**Conclusion:**

Interactive dashboards show promise for supporting safe and effective prescribing in general practice, but current evidence is inconclusive. Future research should focus on developing core outcome sets to facilitate future meta-analyses of effectiveness as well as optimizing their implementation and understanding how to sustain user engagement.

**Key messages**

- Improved primary care data enables real-time comparative feedback to prescribers.
- A limited number of studies evaluating the effectiveness of interactive dashboards were identified.
- Prescribing outcomes were heterogeneous across patient, consultation, and practice levels.
- There is a need for standardized core outcomes sets.

## Introduction

Prescribing is the most common healthcare intervention and advances in therapeutics have improved the lives and life expectancy of many people living with chronic illness [1]. However, alongside these improvements, there has been an increase in potentially inappropriate prescribing (PIP) which has potential negative consequences for individuals, society, and healthcare systems [2]. Prescribing for older patients with multiple chronic illnesses is particularly challenging as prescribers must contend with both potential drug–drug and drug–disease interactions [3]. The reasons for PIP are complex and multifaceted and include systems failures, particularly at the interface between primary and secondary care, individual patient factors such as increasing age and multimorbidity, clinician factors, such as knowledge and attitudes and broader societal attitudes about the roles and benefits of medicines [4]. To both measure and address this, a variety of explicit criteria have been developed for use in both research and clinical practice [5]. These measures tend to focus on highly prevalent PIP or those with the potential for significant harm. More recently there has been a shift towards identifying and addressing low-value prescribing, which on a population level can result in harm both directly (e.g. experiencing adverse effects from medicines that are not providing benefit) and indirectly (e.g. contributing to non-adherence and through the opportunity cost of other cost-effective interventions not resourced due to spending on low-value medicines) [6]. The application of explicit measures of medication appropriateness has been demonstrated to be effective at improving prescribing and has the advantage of being relatively reproducible, reliable, and easy to apply to large numbers of people [7].

With recent advancements in electronic healthcare records (EHR) and prescribing, it is now possible to apply explicit criteria to routine prescribing or dispensing datasets. OpenPrescribing.net is a prominent example, where the vast amount of anonymous prescription data published by NHS England each month is analysed and presented on a web platform to allow for comparative benchmarking between practices [8]. However, when using publicly available anonymous data, only a subset of explicit criteria can be applied as patient-level data such as age and co-morbidities are not included. In addition, it is challenging for clinicians to identify and act on individual instances of sub-optimal prescribing. One approach to combine both audit and feedback and clinical decision support (both of which have been identified as effective methods to improve prescribing [9, 10]), while maintaining anonymity, has been to embed code within practice systems and export aggregate-level data [11]. This aggregated data can then be fed back to individual practices in the form of interactive dashboards and allow for comparative benchmarking. This approach ensures that individual patient identities are protected while allowing practices to compare their performance, identify areas for improvement, and act on individual instances of high-risk prescribing. In most countries with developed primary care systems, primary care clinicians are responsible for the majority of prescribing; both initiating new medicines and continuing those started in secondary care [12]. Thus, interventions implemented in primary care have the potential for broader, system-wide impact.Given advancements in data infrastructure in general practice and the need to address both high-risk and low-value prescribing, this systematic review aimed to explore the characteristics and effectiveness of interactive dashboard interventions on prescribing outcomes in general practice with the additional goal of informing future intervention development and e-prescribing infrastructure.

## Methods

The methods have been described previously in our published protocol [13]. This systematic review was prospectively registered on PROSPERO (CRD42023481475), conducted in line with guidance set out in the Cochrane Handbook for Systematic Reviews of Interventions [14], and reported in adherence to PRISMA statement, Supplementary Appendix S1[15].

### Data sources and search strategy

A systematic literature search was conducted 22 November 2023 in the following databases; PubMed, EMBASE, MEDLINE (OVID), PsycINFO (EBSCOhost), CINAHL (EBSCOhost), Scopus, and the Cochrane Library (OVID). This was supplemented by grey literature searches in OpenGrey, CADTH Grey Matters, and the International Clinical Trials Registry Platform (ICTRP) as well as backward and forward citation chasing using an automated citation chaser [16]. There were no restrictions placed on language or year of publication. Search terms included keywords to capture the intervention (e.g. “interactive dashboard,” “clinical audit,” “medical audit,” “benchmarking,” “data visualization”) the population (e.g. “general practitioner,” “primary care*”), and the outcomes (e.g. “PIP,” “prescribing”). See Supplementary Appendix S2 for electronic search reports.

### Eligibility criteria

All interventional designs were eligible for inclusion including randomized controlled trials (RCTs) (e.g. cluster RCTs, step wedged RCTs and individually randomized RCTs) and non-randomized interventional studies for example interrupted time series (ITS) design and controlled before and after studies as recommended by the Cochrane Effective Practice and Organisation of Care (EPOC) group, see Supplementary Appendix S3 for a summary of inclusion and exclusion criteria. The population of interest was primary care prescribers including non-medical prescribers working in primary care (e.g. pharmacists). An interactive dashboard was defined as a platform designed to provide ongoing feedback of real-time (defined as no older than one year) prescribing data in a visual format and that allowed for comparative benchmarking against peers or a set standard. A true interactive dashboard allows direct manipulation of data with visual analytic tools, however, studies that did not have an interactive element but provided feedback of multiple parameters and/or configurations from the dataset were also included. Simple clinical decision support interventions or audit and feedback interventions that did not give longitudinal and ongoing feedback of real-time data were both excluded. Multi-faceted interventions that included interactive dashboards alongside other components such as education, clinical decision support, or targeted behavioural change strategies were included. The outcome of interest was any prescribing-related outcome measure such as explicit prescribing criteria or prescribing rates where a higher rate is described as reflecting lower quality (e.g. antibiotic, benzodiazepine, or opioid use).

### Study selection and data extraction

Identified records were uploaded to Covidence and independently assessed for inclusion based on title and abstract and then full-text papers by two researchers (CMC, PM, AM, AW, FM), blinded to each other’s decisions, with disagreement resolved by consensus. Data was extracted independently by two researchers using a purposely developed data extraction tool in Covidence (CMC, PM), see Supplementary Appendix S4 for a list of all data points extracted. Methodological quality assessment was assessed independently by two reviewers using the Cochrane EPOC risk of bias tool (CMC, PM) [17].

### Analysis

The Template for Intervention Description and Replication (TIDieR) checklist [18] was used as a framework to narratively summarize interventions. For multi-faceted interventions this framework was utilized to describe the interactive dashboard component alone. We categorized effectiveness based on intervention type (for example if there was a truly interactive component to the dashboard or whether the dashboard was part of a multi-faceted intervention), outcome of interest (e.g. high-risk prescribing or antibiotic prescribing rates), and study design. A meta‐analysis using a random‐effects model was performed where at least two studies were comparable in terms of participants, study design, and outcomes. If the desired direction of the effect differed across outcomes, the measure of association (e.g. odds ratio) was inverted to ensure comparability. Heterogeneity across studies was assessed using the *I*² statistic, with an *I*² value greater than 50% considered indicative of substantial heterogeneity. Although 14 studies were identified, a funnel plot was not performed due to the heterogeneity in study design and outcomes. Instead, a narrative assessment was conducted, acknowledging the potential limitations in detecting publication bias.

## Results

### Search results

**Figure 1.**
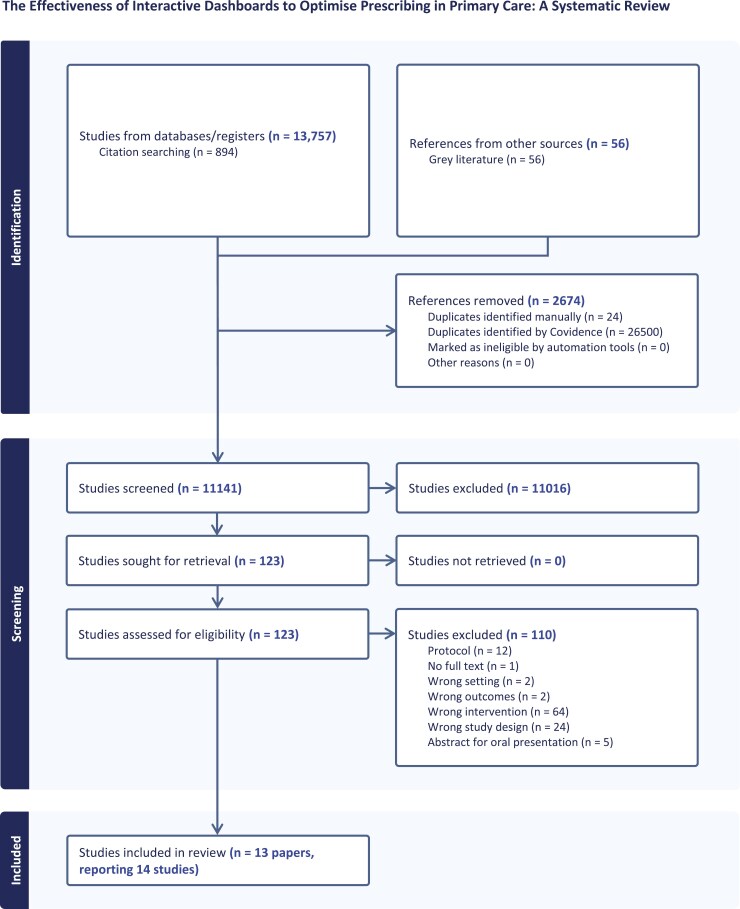
*Study flow diagram.* PRSIMA diagram [15].

A total of 13 757 records were identified from database and citation searching and a further 56 from other sources. Following deduplication, 11 141 records were screened, with 123 full texts assessed for eligibility, and 14 studies, reported in 13 different papers, were included in the review (Fig. 1). See Supplementary Appendix S5 for a table of the excluded studies from full-text review and their reason for exclusion.

### Characteristics of included studies

Of the 14 included studies, three were ITSs [19–21], one was a controlled before and after study [22] and 10 were RCTs [23–31], Supplementary Appendix S6 Characteristics of Included Studies. Six of the RCTs (two of which were reported in the same paper) were a cluster design [24, 26, 28–31]. Two RCTs that targeted individual physician antibiotic prescribing were individually randomized at the physician level [23, 27] and the final RCT was a stepped wedge design where six clusters were sequentially allocated to the intervention every month [25]. One paper described randomly assigning practices to one of two parallel cRCTs [29]. In each cRCT, the control group in one trial served as the intervention group in the other, leading to a total of four distinct studies reported in the same paper. These studies were conducted simultaneously with the same intervention but focussed on different outcomes. However, only two of these four studies were considered relevant for this systematic review, as the outcomes of the other two were not relevant. Therefore, both of the relevant studies from this paper were included in this review [29]. In seven of the included studies, one ITS and six RCTs, participants (either individual prescribers or practices) were not aware of their participation in the study, as the interventions were implemented at a system level or as part of routine policy changes [20, 23, 24, 26–29]. In two additional studies, it is unclear whether practices/physicians were recruited and consented [19, 25] and in the final four studies practices from a defined region were invited to participate [21, 22, 30, 31]. The number of participants included in the individual studies ranged from 12 to 1401 practices and 43 to 3426 physicians. None of the included studies recruited individual patients.

Eight studies focussed on antibiotic prescribing and used aggregated data, for example, the rate of antibiotic prescriptions per 100 consultations or the proportion of all antibiotics that were broad-spectrum [19, 21, 23–25, 27, 30, 31]. The remaining six studies focussed on PIP [20, 22, 26, 28, 29]. Five of these reported the number of patients potentially at risk and affected. The remaining study reported the mean number of patients per practice with an inappropriate bronchodilator prescription but did not provide the numerator and denominator [28]. See Supplementary Appendix S6 for a summary of the characteristics of included studies.

### Characteristics of interventions

The characteristics of the interactive dashboards of the included studies are described based on the TIDieR checklist in Table 1 [18]. The tailoring and modification components of this framework were generally not applicable as these are often more relevant to implementation programs, where interventions may need to be adapted to fit specific contexts or populations [18]. The interventions identified in this systematic review were all designed for use within the context in which they were implemented. Only one study reported a modification where antibiotic prescribing feedback was initially based on the dispensing claim’s date, but in the second year of the study, this was modified to reflect the actual prescription date [27]. Five of the 13 interventions included had a true interactive component where the user could directly manipulate their data and had unlimited access to the dashboard within a defined period of time [19, 20, 22, 24, 30]. The remaining seven interventions all provided prescribers with longitudinal access to relatively real-time data. Seven of the twelve interventions included multi-faceted interventions, where the interactive dashboard was part of a broader program [19, 20, 24–26, 29, 31]. Two of these were three-arm cRCTs where one of the arms received an additional behavioural change component [24, 26]. Two dashboards also alerted prescribers to individual patients with high-risk prescribing/inadequate blood-test monitoring [20] or inappropriate bronchodilator prescriptions [28].

**Table 1.** Characteristics of interventions

Study ID Intervention name	Multi-faceted?	Why?	What?	Who provided it?	How and where?	When and how much?	How well?
**Interventions with a true interactive component**							
***Curtis 2021*** [24]	Yes, arm 3 feedback included one page theory informed behavioural change component.	To increase engagement with prescribing data and change prescribing.	Link to specific metrics on publicly available prescribing dashboard with either plain or specific feedback.	Dashboard publicly available at openprescribing.net. Link and feedback provided by research team.	Link and feedback sent by post, email and fax.	May—July 2018, monthly for 3 months	Dashboard engagement measured using Google analytics.
***Davidson 2023, CHOSEN*** [19]	Yes, web platform which included webinars on antimicrobial stewardship, how to use the dashboard and resources for patients.	To reduce inappropriate antibiotic prescribing	Web-based platform that provided interactive visualizations of abx prescribing rate that was updated on a monthly basis. Data could be explored by visit type, primary location and provider. Monthly and yearly trends were displayed with an overall rate and a target rate.	Research team	Web-based dashboard which could be accessed during study period.	Accessible at any time from February 2018	No mention of usage analytics
***deLusignan 2021*** [22]	No	To reduce inappropriate metformin and aspirin prescribing	Dashboard to allow GPs monitor their prescribing of metformin for patients with eGFr < 30 and > 30 > 40 and aspirin for patients with T2DM who do not have CKD/CVD	Research team	Dashboard built directly into EHR	Metformin dashboard available from April-July2019. Aspirin dashboard available from July -Oct 2019	Not measured
***Linder 2010*** [30]	No	To reduce antibiotic prescribing for RTIs	Dashboard to allow GPs compare their antibiotic prescribing for RTIs to colleagues and national benchmarks.	Research team	Dashboard built directly into EHR	Accessible anytime from November 2006 until August 2007	28% used the dashboard at least once. (Over half of whom used it only once.)
***Peek 2020, SMASH*** [20]	Yes, clinical pharmacists trained to deliver the intervention in partnership with general practice staff. Feedback included educational material about each of the included indicators.	To reduce hazardous prescribing and inadequate blood-test monitoring	Interactive dashboard that provided feedback on prevalence of each hazardous prescribing and inadequate blood-test monitoring indicator with comparisons to the CCG average. Data was displayed in both tabular and graph format. Individual patients at risk were also listed by NHS number.	NHS- phased roll out based on NICE guidance	Web-based interactive dashboard	Data updated on a daily basis and available to practices from April 2016.	Not measured
**Interventions without a true interactive component**							
***Aghlmandi 2023*** [23]	No	To improve abx prescribing rates amongst higher rate prescribers.	Graphic abx prescribing report comparing prescribing rates to the previous 3 months and to peers.	Research team using insurance claims data	Email to the physician	Quarterly email from December 2017-September 2019	Not measured
***Dutcher 2021*** [25]	Yes, once-off educational session on appropriate prescribing for RTIs	To reduce antibiotic prescribing for RTIs	Graphic report displaying overall antibiotic prescribing rate and inappropriate RTI abx prescribing rates with comparisons to the average and best performing practices.	Research team	Automatically generated reports with data extracted from EHR—emailed to practice	Monthly feedback from October 2017—October 2018	Not measured
***Gerber 2013*** [31]	Yes, once-off educational session providing updates on antimicrobial prescribing guidelines	To reduce (off-guideline) broad spectrum antibiotic prescribing	Personalised audit and feedback of guideline-based antibiotic prescribing rates for the individual, the individual’s practice and the network	Research team	Emailed to individual clinicians within each practice	June 2010—June 2011, every four months	Not measured
***Guthrie 2016, EFFIPPS*** [26]	Yes, all three arms were emailed educational material. Arm 3 feedback included a 1-page theory informed behavioural change component.	To improve the safety of primary care prescribing	Graphic report comparing the practice’s high risk prescribing against the rate achieved by the 25th percentile of Scottish practices with the most optimal rates in the year before feedback started.	NHS Scotland Information Services Division	Email to practice	Five emails sent quarterly from June 2012—June 2013	Not measured
***Hemkens 2017*** [27]	No	To reduce antibiotic prescribing	Graphs showing monthly trend in the physician’s abx prescribing rate per 100 consultations and compared to the adjusted peer average.	Research team	Posted report with access code to the study website for more detailed feedback	8 postal feedbacks between October 2013 and July 2015	Not measured
***MacBride-Stewart 2022*** [28]	No	To reduce inappropriate prescriptions of bronchodilator inhalers.	Tables comparing inappropriate SABA or LABA prescriptions with the rest of the health board. List of individual patients identified as receiving inappropriate prescriptions.	Report developed by research team but also had signatures from three key lead clinicians in the health board.	Emailed to secure practice address and copied to the practice’s prescribing support team pharmacist.	Three times: July 2015, February 2016 and August 2016.	69.2% practices discussed feedback with team. 80.4% reviewed ≥1 patient record. 77.6% flagged ≥ 1. patient record. 62.6% consulted with ≥ 1 patient.
***Soucy 2024, OPEN Stewardship*** [21]	No	To reduce antibiotic prescribing rate	Personalised prescribing reports displaying overall abx prescribing rate, rates for specific conditions and comparisons against the average and 25th percentile for other intervention participants	Research team	Report emailed directed to physicians	Quarterly for 9 months	Not measured
***Willis 2020, ASPIRE*** [29]	Yes, practices were offered two training sessions by pharmacist, CDSS prompts for risky prescribing and tools to search for high-risk patients	To target barriers to change prescribing for four quality indicators.	Practice specific report displaying temporal trends for each of the trial indicators in number and graph form. Practices were also ranked and compared to others in the CCG.	Research team	Reports sent by post and email to the practice	Quarterly from May 2015-March 2016	Implementation will be assessed via process evaluation

*Although 14 studies are included in this review, two studies were reported in the same paper and used the same study processes and intervention type but focussed on different outcomes. For the purpose of describing the intervention, these two studies are considered as one.*

*Abx; antibiotic, EHR; electronic health record, eGFR; estimated glomerular filtration rate, CKD; chronic kidney disease, CVD; cardiovascular disease, CCG; clinical commissioning group, NHS; National Health Service, NICE; National Institute for Clinical Excellence, RTI; respiratory tract infection, SABA; short-acting beta-agonist, LABA; long-acting beta agonist, CDSS; clinical decision support-system*.

### Risk of bias in included studies

**Figure 2.**
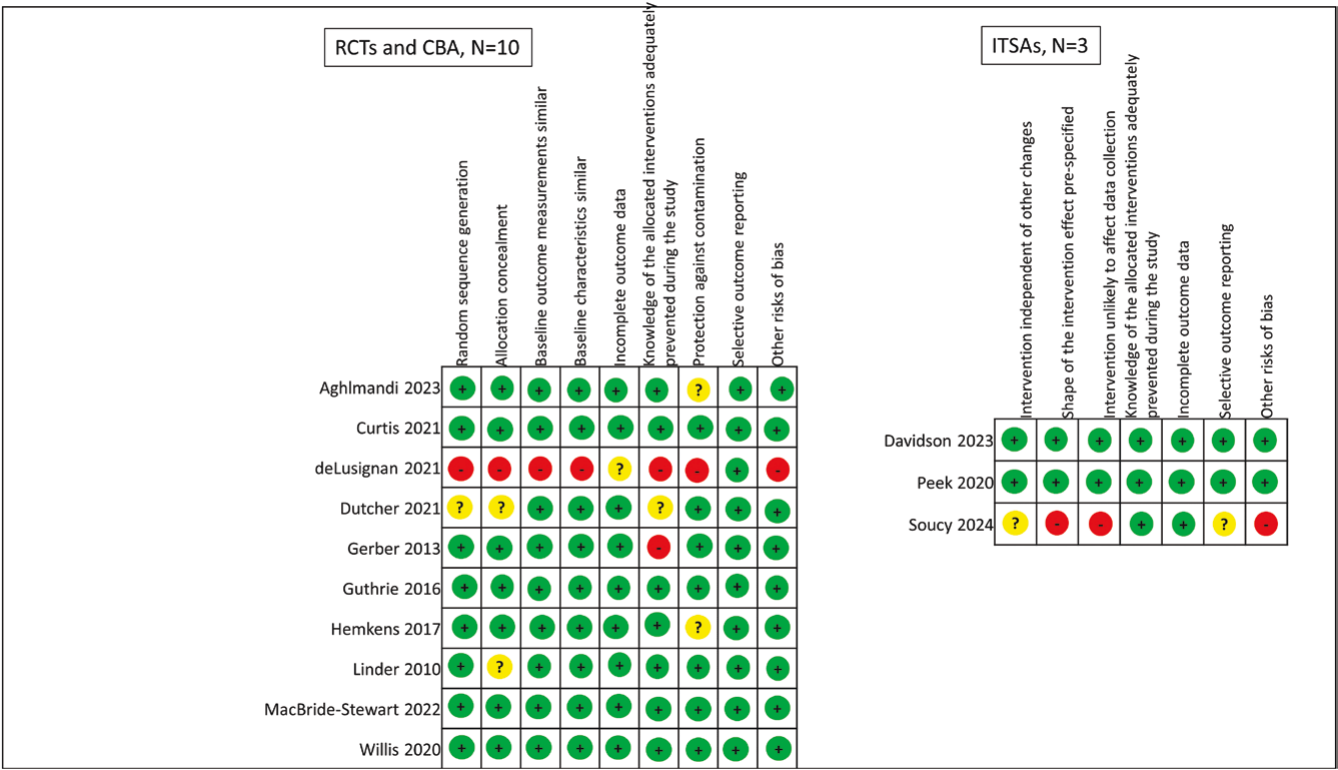
*Cochrane effective practice and organization of care risk of bias summary* [17]. The two studies reported within the one paper were considered as one for the risk of bias assessment as they had the exact same study processes.

Overall eleven of the 13 included papers had a low risk of bias [19, 20, 23–29]( Fig. 2). See Supplementary Appendix S7 for risk of bias graphs summarizing the risk for each EPOC criterion. One ITS had a moderate risk of bias [21], where there were significant losses to follow up amongst Canadian physicians that were inadequately addressed, in addition, the COVID-19 pandemic was likely to have influenced antibiotic prescribing during the intervention period. There was also insufficient information on prevention of knowledge of allocated interventions and crude post-intervention prescribing rates were not presented which limited transparency [21]. The included controlled before and after study had a high risk of bias, by virtue of its design [22]. In addition, there was a significant improvement in inappropriate aspirin prescribing prior to intervention implementation, this was clearly reported by the authors but unexplained [22], given this finding the intervention effect was not considered significant for the purpose of this review.

### Effectiveness of interventions

Overall, seven of the 14 included studies, two ITSs [19, 20] and five RCTs [25, 26, 28, 29, 31] demonstrated a significant effect on prescribing-related outcomes, Table 2. When exploring the intervention effect by outcome measure, three of the eight studies that targeted antibiotic prescribing had a significant effect [19, 25]. One of these had a true interactive component where prescribers had ongoing access to real-time data [19] and all three were multi-faceted interventions that included an educational component [19, 25]. One was a prospective ITS where practices after the pre-intervention and wash-out periods received access to a web platform that provided educational material and interactive dashboards [19]. Data was updated on a monthly basis and displayed as graphs illustrating monthly and yearly trends in the antibiotic prescribing rate that could be explored by visit type, primary location, and provider [19]. The effect of the intervention was explored by provider type (paediatric, internal medicine, family physicians, and urgent care) with the family physician rate reported here, Table 2. The largest effect size was seen for this group, although significant results were seen in all groups [19]. Another study that showed a significant effect on antibiotic prescribing was an RCT that focussed on inappropriate antibiotic prescribing for respiratory tract infections [25], with the outcome measured at the visit level (i.e. the proportion of respiratory tract infection visits that resulted in the prescription of an antibiotic). Participating practices received an educational package and monthly reports by email which displayed the overall antibiotic prescribing rate and the rates of inappropriate antibiotic prescribing for defined respiratory tract infections, both of which were compared to the average and best-performing practices [25]. The final study that demonstrated a significant effect on antibiotic prescribing was an RCT that focussed on inappropriate broad-spectrum antibiotic prescribing by paediatric primary care clinicians [31]. Participating clinicians received individualized feedback on their rate of broad-spectrum antibiotic prescribing for various different respiratory tract infections [31]. The remaining five studies that targeted antibiotic prescribing failed to show an effect on antibiotic prescribing rates [21, 23, 27, 30] or broad-spectrum antibiotic prescribing [24].

**Table 2.** Results of included studies

Lead author, year, country	Outcome description	Effect size
*** Randomised controlled trials***		
Aghlmandi (2023, Switzerland) [23]	Abx prescribing rate per 100 consultations during the second year of the intervention	ARR –0.1% (95% CI, –1.2% to 51.0%)
Curtis (2021, UK) [24]	Percentage of abx that are broad spectrum comparing those with (a) plain feedback versus no feedback, and (b) behavioural impact with feedback versus plain feedback.	(a) Coefficient − 0.31%, (95% CI − 0.7% to 0.1%) (b) Coefficient 0.41%, (95% CI 0.007% to 0.800%)
Dutcher (2021, USA) [25]	Proportion of RTI visits with abx	OR 0.47 (95% CI 0.45 to 0.48)
Gerber (2013, USA) [31]	Rates of broad-spectrum antibiotic prescribing for bacterial and RTIs	Difference of differences, 6.7%; (p = 0.01 for differences in trajectories)
Guthrie (2016, UK) [26]	Proportion of patients included in one or more of the 6 criteria who received a high-risk prescription comparing those with (a) plain feedback versus no feedback, and (b) behavioural impact with feedback versus no feedback	(a) OR 0.88 (95% CI 0.80 to 0.96) (b) OR 0.86 (95% CI 0.78 to 0.95)
Hemkens (2017, Switzerland) [27]	Antibiotic prescription rate measured as DDD per 100 consultations	ARR 0.81% (95% CI -2.56% to 4.30%)
Linder (2010, USA) [30]	Antibiotic prescribing rate for RTIs	OR 0.97 (95% CI 0.7 to 1.4)
MacBride-Stewart (2022, UK) [28]	Mean number of patients with an inappropriate SABA or LABA prescription	Mean difference -3.7 (95% CI -5.3 to -2.0)
Willis (2020, UK) [29]^b^	Proportion of patients with at least 1 high-risk NSAID or anti-platelet indicator	OR 0.815 (97.5% CI 0.67 to 0.99)^a^
Willis (2020, UK) [29]^b^	Proportion of patients with AF prescribed appropriate anticoagulation therapy^b^	OR 0.902 (97.5% CI 0.75 to 1.09)
*** Controlled before and after study***		
deLusigan (2021, UK) [22]	Proportion of patients incorrectly prescribed (a) metformin and (b) aspirin	(a)^c^ (b) OR 0.44 (95% CI 0.27 to 0.72)^d^
*** Interrupted time series***		
Davidson (2023, USA) [19]	Percentage of total family physician visits with abx prescription	Pre–post relative difference in rates -20.4 % Level change β Coefficient − 7.95 (95% CI − 11.05 to − 4.85)
Peek (2020, UK) [20]	Prevalence of exposure to (a) any potentially hazardous prescribing (10 indicators) and (b) any inadequate blood-test monitoring (2 indicators) among patients with risk factors for such prescribing and monitoring 12 months after intervention start	(a) ARR -0.96% (95% CI -1.12% to -0.79%) (b) ARR − 2.85% (95% CI − 5.68% to 0.71%)
Soucy (2024, Israel/ Canada) [21]	Proportion of total visits with abx prescription	OR 1.01; 95% CI 0.94 to 1.07)

^a^Results presented as 97.5% CI, converted to 95% for meta-analysis below.

^b^Odds ratio was inverted to for comparison to other PIP measures.

^c^No inferential statistics for the first outcome measure, proportion with inappropriate metformin prescriptions, as number were very low (< 10).

^d^Unaccounted changes in the same direction of the intervention effect seen prior to implementation of the intervention.

*ARR; absolute risk reduction, OR; odds ratio, Abx; antibiotic, RTI; respiratory tract infection, DDD; defined daily dosage, SABA; short-acting beta agonist inhaler, LABA; long-acting beta agonist inhaler, AF; atrial fibrillation, NSAID; non-steroidal anti-inflammatory drug*.

Six studies (reported in five papers) explored the effect on PIP for other drugs groups (see Supplementary Appendix S8 for a list of all the indicators included in these studies) and four of these demonstrated a significant effect [20, 26, 28, 29]. Three of these four were part of multi-faceted interventions that included either a clinical decision support or educational element [20, 26, 29], and only one was a true interactive dashboard where prescribers had ongoing access to real-time data [20]. The four studies that demonstrated a significant effect on PIP included one ITS and three RCTs. The ITS explored the effect of the routine roll-out of the SMASH intervention that provided feedback on the prevalence of each hazardous prescribing and inadequate blood-test monitoring indicator with comparisons to the local area (clinical commissioning group) average [20]. There was a significant effect on high-risk prescribing (ARR -0.96%, 95% CI -1.12% to -0.79%) but not on inadequate blood-test monitoring [20], however, there was a significant effect seen on the latter outcome at 24 weeks follow-up. In addition, the largest reductions in high-risk prescribing were seen in practices with higher baseline rates and by 12 months follow-up there was significantly reduced inter-practice variation in the rates of PIP and inadequate blood test monitoring [20]. The remaining three studies demonstrating a significant effect on PIP were RCTs where feedback with comparative rates of each of the included indicators were emailed to the practice every quarter [26, 28, 29].

Of the two studies that did not demonstrate an effect on PIP, one was a controlled before and after study that had a high risk of bias [22]. Although the authors reported a significant effect on inappropriate aspirin prescribing there was an unexplained significant improvement in this indicator during phase one of the study prior to the implementation of the aspirin dashboard [22]. In addition, no rates or inferential statistics for the effect of the dashboard on inappropriate metformin prescribing were reported as there were only 8 patients prescribed this medicine at follow-up [22]. The other study that did not demonstrate an effect on PIP was a cRCT that explored the effect on inappropriate anticoagulant omissions for atrial fibrillation [29].

With respect to the intervention components six of the seven studies that had multi-faceted interventions showed a significant effect [19, 20, 25, 26, 29, 31], compared to one [28] of the six studies that just had the interactive dashboard component. Two cRCTs included clinical decision support elements where individual patients were identified within the dashboard (hazardous prescribing/inadequate blood test monitoring or inappropriate bronchodilator prescribing) and both had a significant effect [28, 29].

**Figure 3.**
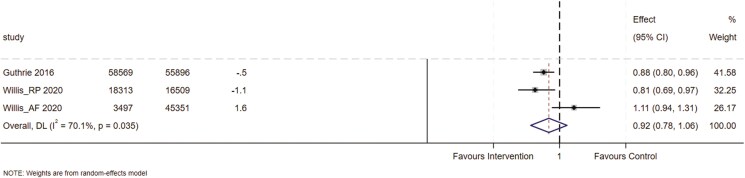
*Forest plot of randomized controlled trials exploring the intervention effect on patient level prescribing appropriateness.* Abbreviations: CI; confidence interval, RP; risky prescribing, AF; atrial fibrillation. Forest plot showing the effects of intervention on prescribing outcomes across included studies. For the two Willis studies, the originally reported 97.5% confidence intervals (CIs) were converted to 95% CIs for consistency. Additionally, for the atrial fibrillation study, the outcome measured was the improvement in appropriate anticoagulant prescribing for atrial fibrillation; the odds ratio (OR) was inverted to align with the direction of effect in the meta-analysis.

Four of the eight included RCTs measured the effect of the intervention on PIP and presented results as proportions and odds ratios with 95% confidence intervals (two of these trials were reported within one paper) [25, 26, 29]. However, one of these studies measured the proportion of inappropriate prescriptions at the visit level [25], the others were at the patient level [26, 29]. Thus the three RCTs that assessed the effect on appropriate prescribing at the patient level were included in the meta-analysis. The overall effect size was OR 0.92 (95% CI 0.78 to 1.06), with significant heterogeneity (*I*^2^ =70.1%, *P* = .04) (see Fig. 3). Two of these studies used composite measures of high-risk prescribing, see Supplementary appendix S9 for grouped analyses by outcome type. This heterogeneity may be explained by differences in outcome measures: the trial on appropriate atrial fibrillation prescribing assessed improvement in prescribing, whereas the other two studies measured the presence of at least one high-risk prescribing indicator.

## Discussion

### Summary of results

This systematic review aimed to explore the characteristics of interactive dashboard interventions designed to support safe prescribing and explore their effect on prescribing-related outcome measures. Given the nature of the intervention, where data are often fed back on a widespread basis and implemented as a policy, quasi-experimental designs, were included. Seven of the 14 included studies demonstrated a significant effect and all of these had an overall low risk of bias [19, 20, 25, 26, 28, 29, 31]. Notably, only three of the eight studies that explored the effect of these interventions on antibiotic prescribing showed a significant effect [19, 25, 31]. It may be that recent campaigns to improve antimicrobial stewardship have already resulted in improvements, leaving less room for further advancements. However, a 2010 study did not see any effect on antibiotic prescribing for acute respiratory infections concluded that antibiotic prescribing rates were high [30]. Four of the five studies looking at PIP showed a significant effect [20, 26, 28, 29]. Potentially inappropriate or high-risk prescribing is more common in complex patients with multimorbidity and polypharmacy [2] and lack of prescriber awareness has been identified as a barrier to addressing this [32]. Thus, the difference in effect seen by outcome measure may be because PIP is often related to clinician oversight, whereas antibiotic prescribing reflects a more direct clinician decision and thus may be less amenable to change. This hypothesis is supported by the fact that both interventions that alerted prescribers to specific instances of PIP had a significant effect [20, 28]. Similar to results presented in other systematic reviews exploring the effectiveness of interventions in addressing inappropriate polypharmacy [33], multi-faceted interventions were more often effective with five of the six multi-faceted interventions demonstrating a significant effect [19, 20, 25, 26, 29]. In summary, interventions targeting PIP, that included multifaceted elements seemed to have a beneficial effect on outcomes.

Audit and feedback are known to lead to small improvements in professional behaviour, as evidenced by a systematic review of 140 studies, including a meta-analysis of 108 comparisons from 70 studies, which found a median absolute increase of 4.3% in healthcare professionals’ compliance with desired practices [9]. Increased frequency of feedback, coupled with explicit, measurable targets and specific action plans, were associated with greater effectiveness [9]. The interventions included in this review all included ongoing feedback of relatively contemporaneous data, with comparisons to specific set targets or comparative benchmarking. There was significant heterogeneity among the results of the three studies included in this meta-analysis, and thus the results should be interpreted with caution. This was likely explained by the differing outcome measure in the study assessing the effect on appropriate coagulation for atrial fibrillation, where the outcome reflected inappropriate omission as opposed to an inappropriate prescription. Other approaches such as educational interventions and clinical decision support have also failed to improve anticoagulation rates for patients with atrial fibrillation [34]. Collaborative, shared decision-making interventions may be needed to address this more complex prescribing decision around reducing the future risk of an adverse event [35]. The two other studies included in the meta-analysis, both of which demonstrated a significant effect, targeted PIP and included an educational component [26, 29], which may have mediated some of the observed effect. The study by Willis et al, which showed a larger treatment effect, also incorporated clinical decision support, which has been shown to have a modest effect on prescribing behaviour [36].

Similar systematic reviews have explored the effectiveness of interactive dashboards on antibiotic prescribing [37] and more generally on medication prescription, adherence, and test ordering [38]. Similar to this review both of these reviews included dashboards that were implemented as part of multi-faceted interventions but unlike our review, the focus for one of these reviews was not restricted to primary care and for the other, the focus was restricted to antibiotic prescribing specifically. Both reviews noted the potential for interactive dashboards to improve clinician behaviour but that there was significant variation in results [37, 38]. This heterogeneity was attributed to potential differences in the setting, the outcomes, and features of the dashboards themselves. Given that both audit and feedback and CDSS interventions are known to have a small but significant positive effect on prescribing, it has been proposed that the focus should now be to systematically test various approaches to the design and development of these interventions, which will include the development of dashboards designed to deliver both audit and feedback and CDSS [39].

### Strengths and limitations

This systematic review included a broad and detailed search strategy including citation chasing and grey literature searches of clinical trial registries to reduce the risk of publication bias. Including quasi-experimental designs meant we captured two large ITSs that explored the implementation of novel interactive dashboards. Given the heterogeneous nature of the outcome measures included different prescribing criteria, measured at different levels (e.g. practice and visit level as well as patient level) it was only possible to perform a meta-analysis for three studies and it was not possible to conduct a funnel plot to formally assess publication bias. The scope of our review was shaped by focussing on feedback mechanisms that align with the functionality of interactive dashboards. While this approach excludes one-off feedback interventions, it allows for a more detailed exploration of tools designed for repeated engagement with contemporaneous data, which are hypothesized to have distinct advantages [40]. This focus also aligns with prior evidence suggesting that repeated feedback may be more effective in driving behaviour change [39], though we acknowledge that findings from recent research indicate variability in this relationship [41].

### Implications for research, policy and practice

This review identified several gaps in the existing literature. Only 14 studies met the inclusion criteria, with just five employing true interactive dashboards and seven were multifaceted. The limited number of studies suggests a need for further research exploring the effectiveness of interactive dashboards designed to optimize prescribing. Of note, all included studies were from high-income settings, mostly North America and Europe meaning the results may not be generalizable outside of these contexts and suggesting that more research is needed from low and middle income countries. A key challenge in conducting the meta-analysis was the heterogeneity of outcome measures. Identifying appropriate outcome measures is a challenge when assessing the effectiveness of interventions such as interactive dashboards designed to improve prescribing quality in primary care settings. Important clinical endpoints such as unplanned hospital admissions or mortality require a sufficient sample size and an adequate follow-up period to identify any potential effect mediated by higher-quality prescribing, which may not be feasible. Thus, composite measures of explicit prescribing criteria are often used as primary endpoints for these studies [42]. There is strong observational evidence that such prescribing is associated with adverse outcomes for patients such as increased mortality, falls, and unplanned hospital admissions [43]. More general prescribing measures include rates of utilization and these serve as a valuable outcome for evaluating the impact of health policy implementation programs aimed at modifying prescribing behaviours, such as reducing high opioid prescription levels [44] or imposing restrictions on drugs with unfavourable cost-effectiveness profiles [45]. However, the outcomes identified for this review were reported at various levels, including the prescription, patient, prescriber, and practice levels. The development of a core outcome set for prescribing-related measures that can be consistently applied across studies utilizing routine prescribing or dispensing datasets may be one way of addressing this and would facilitate more meaningful comparisons and benefit future meta-analyses. Future research should also investigate how different feedback features such as frequency, content, and format influence both effectiveness and engagement, and whether there is an optimal level of engagement required for behaviour change. This will necessitate systems capable of providing both ongoing feedback and measuring user engagement through detailed use analytics, the latter of which was notably absent in most studies included in this systematic review. Finally, routine evaluations of policies that utilize this data to optimize prescribing are essential. However, it is important to bear in mind that explicit measures of appropriateness are typically developed by expert consensus and do not account for individual patient characteristics. Thus, these evaluations should include an assessment of the effect on important clinical outcomes in addition to prescribing-related outcome measures. Given rising concerns around equity in access to medicines and overprescribing [46], evaluating the cost-effectiveness of these interventions will also be important. Systematic and standardized evaluations would provide valuable insights into the effectiveness of policy interventions aimed at optimizing prescribing, thereby enhancing clinical outcomes and health system efficiency.

## Conclusion

Interactive dashboards have the potential to support safe and effective prescribing in primary care, although current evidence is inconclusive. Multi-faceted interventions that target high-risk prescribing are more likely to be effective. Future research should focus on developing core outcome sets to facilitate future meta-analyses of effectiveness as well as optimizing their implementation and understanding how to sustain user engagement. To support their implementation, it is essential to establish the necessary data infrastructure within primary care systems. With advancements in data infrastructure and analysis, these interventions could have a significant impact if implemented at scale.

## Supplementary Material

cmaf036_suppl_Supplementary_Figures_S1-S9

## Data Availability

No new primary data were collected for this study, as the review synthesizes publicly available data from published studies. All data generated or analysed during this study are included in this published article and its supplementary information files. This includes the search strategies used for each database, details of the included studies, and any additional data extracted during the review process. The study protocol is available as an open-access publication and can be accessed directly at https://hrbopenresearch.org/articles/7-44.
